# Bone marrow mesenchymal stem cells derived exosomal Lnc TUG1 promotes bone fracture recovery via miR-22-5p/Anxa8 axis

**DOI:** 10.1007/s13577-023-00881-y

**Published:** 2023-03-23

**Authors:** Wei Li, Lihong Li, Rui Cui, Xiaoqing Chen, Haifeng Hu, Yuyu Qiu

**Affiliations:** 1 Department of Clinical Laboratory, China Japan Union Hospital of Jilin University, Changchun, 130033 China; 2 Department of Orthopedics, Shandong Provincial Hospital Affiliated to Shandong First Medical University, No.324, Jingwuweiseven Road, Huaiyin District, Jinan, 250014 Shandong Province China; 3 Shandong First Medical University (Shandong Academy of Medical Sciences), No.6699, Qingdao Road, Huaiyin District, Jinan, 271016 Shandong Province China

**Keywords:** lncTUG1, Bone fracture recovery, miR-22-5p, Anxa8

## Abstract

Bone fracture healing is a complex physiologic process that involves changes in the expression of several thousand genes. Long noncoding RNAs (lncRNAs) may have critical biological roles in this process. The objectives of the present study were to determine whether BMSC-derived exosomal lncTUG1 can enhance osteogenic differentiation and thereby promoting bone fracture recovery and to investigate its potential mechanisms of action. Bone marrow mesenchymal stromal cells were isolated from mice and cultured for the following experiments. After adipogenic and osteogenic differentiation induction, Oil Red O, alizarin red S, and alkaline phosphatase staining solutions were applied to confirm the formation of lipid droplets and calcium nodules. Western blotting analyses, real-time reverse transcription PCR assays, luciferase reporter were performed to confirm relative RNA and protein expressions and luciferase activities of transfected cells. RNA pull-down and RNA immunoprecipitation assays were also carried to verify the interaction between lncTUG1 and miR-22-5p. Additionally, a mouse model of closed femoral fractures was generated to evaluate the in vivo effect of increased lncTUG1 on fracture healing. BMSC-derived exosomal lncTUG1 enhanced the activity of osteoblasts. Overexpression of miR-22-5p reversed the osteopromoting effect of increased lncTUG1. The knockdown of Anxa8 reversed the osteogenic effect of miR-22-5p inhibitors, indicating an interaction between Anxa8 and miR-22-5p. Upregulation of lncTUG1 could promote the fracture recovery in vivo. In conclusion, the present study highlights the functional importance of BMSC-derived exosomal lncTUG1 in the process of bone fracture recovery.

**Supplementary Information:**

The online version contains supplementary material available at 10.1007/s13577-023-00881-y.

## Introduction

Fracture healing is a highly orchestrated, regenerative process initiated in response to injury [1]. It follows a definable temporal and spatial sequence and involves the coordinated action of many cells, proteins, and the expression of hundreds of genes working towards restoring cellular composition, structural integrity, and biomechanical function of the bone. Any deficit in the repair process may lead to abnormal or delayed fracture healing and predispose patients to complications. With the advances made in the biology of bone healing, extensive research is ongoing to elucidate the role of different molecules and their interactions in the process of fracture callus formation and resolution [2]. Emerging evidence shows that long noncoding RNAs (lncRNAs) can be critical to the maintenance of bone homeostasis [3, 4].

LncRNAs are currently defined as transcripts with more than 200 nucleotides in length [5]. In broad terms, lncRNAs encompass a large and heterogeneous collection of RNA transcripts that differ in their biogenesis and genomic origin, including enhancer RNAs, snoRNA hosts, intergenic transcripts, and transcripts overlapping other transcripts in either sense or antisense orientation. LncRNAs are often detected in nucleoli, chromatin speckles, and paraspeckles, but they can also be found in mitochondria, ribosomes, extracellular membranes, and exosomes [6]. The expression level of lncRNAs, on average, is lower than protein-coding genes. However, in general, their expression pattern resembles that of protein-encoding genes and highly depends on developmental stages, cell and tissue types, and disease states. Despite a lack of protein-coding potential, lncRNAs are involved in a wide range of biological functions; they act both in *cis*, at the site of transcription, or in *trans*, at distantly located genes, through diverse molecular mechanisms, most of which require the interaction with one or more RNA-binding proteins [7].

A lncRNA–lncTUG1–was recently detected in the femoral metaphysis of mice. There is evidence suggesting that lncTUG1 may promote osteoblast differentiation via acting as a microRNA (miRNA) sponge to upregulate Runx2 [8]. Our previous sequencing analyses showed a high expression of miR-22-5p in the exosomes derived from rat bone marrow marrow mesenchymal stromal cells (BMSCs). Rat, mouse, and human miR-22-5p have high homology. This miRNA was reported to have a regulatory effect on osteolysis [9]. According to databases’ prediction, lncTUG1 and miR-22-5p have binding sites, indicating that they may interact with each other. In addition, we searched six databases and identified a downstream-targeted gene of miR-22-5p, Anxa8. The expression of Anxa8 was reported to be important in the regulation of osteoclast differentiation [10]. Therefore, the aim of the present study was to investigate whether BMSC-derived exosomal lncTUG1 can enhance osteogenic differentiation and thereby promoting bone fracture recovery via miR-22-5p/Anxa8 axis.

## Materials and methods

### Isolation and culture of mouse BMSCs

BMSCs were isolated from bone marrow cells of 8-week-old male mice and cultured as described previously [11]. In brief, the mice were terminated by cervical dislocation and sterilized with 75% alcohol, then the femora and tibias were separated and collected. The bones were incubated in minimal essential medium (MEM) α10%FBS with 1 mg/ml Collagenase II for 90 °C at 37 °C with shaking. The cells were rinsed and then incubated in αMEM10%FBS medium at 37 °C with 5%CO_2_ for 3 days, during which the cells were split a couple of times for proliferation. Nonadherent cells were removed by changing the medium, and adherent cells were obtained. The medium was changed every 3 days. BMSCs at Passage 3 cells were randomly selected and used for subsequent experiments.

### Extraction of exosomes from mouse BMSCs

When cell confluence reached 80%, the 3rd passage BMSCs were rinsed twice by phosphate-buffered saline (PBS) and cultured for 48 h with serum-free Dulbecco’s Modified Eagle Medium. The supernatant was collected and centrifuged at 300 g, 2000 g, and 10,000 g to remove dead cells and cell debris. Then the supernatant was collected and centrifuged at 10 × 10 g and again at 1.0 × 10 g to remove the impure protein to obtain the exosomes precipitation. The exosomes precipitation was suspended by PBS. The bicinchoninic acid protein method was used to detect the concentration of exosomes, and the exosome samples were preserved at − 80℃.

### Oil red O staining

BMSCs and osteoblasts were co-cultured in an adipogenic differentiation medium, and the oil red O staining was applied to detect lipid droplets. In brief, 0.05 g of Oil Red O powder (00,625, Sigma-Aldrich) was added to 99% propanediol (134,368, Sigma-Aldrich) to prepare Oil Red O dyes. After removing the culture medium, the cells were washed with 1 mL of PBS twice. Next, the cells were fixed with 4% paraformaldehyde in PBS for 15 min at room temperature. After removing the solution, the cells were washed with diH2O three times, and 0.5% Oil Red O was added to incubate the cells for 1 h. After incubation, the cells were washed with 70% ethanol three times and analyzed under a microscope.

### Alizarin red S

Alizarin Red S was (TMS-008, Sigma-Aldrich) performed according to the manufacturer’s protocol. The cells were fixed incubating in ice-cold 70% ethanol for 5 min at room temperature. After aspirating the alcohol, the cells were rinsed twice, 5 min each time, with distilled water. Then, 1 mL of 2% Alizarin Red S Stain solution was added to each well and incubated with the cells for 3 min at room temperature. Then the solution was removed and the cells were washed with distilled water five times. The pH value of the Alizarin Red S solution was adjusted to 4.1–4.3 with ammonium hydroxide prior to the procedure.

### Cell transfection

miR-22-5p mimic, mimic negative control (mimic-NC), miR-22-5p inhibitor, inhibitor negative control (inhibitor NC) were purchased from GenePharma (Shanghai, China) and transfected into the cells using Lipofectamine™ RNAiMAX Transfection Reagent (catalog# 13778075, Invitrogen) according to the manufacturer's instructions.

### Western blotting

The cells were lysed in RIPA buffer supplemented with proteinase and phosphatase inhibitors (KeyGen). Total protein concentration was measured using an Enhanced BCA Protein Assay Kit (Beyotime, P0010S). Equal amounts of protein lysates were electrophoresed on 10% SDS-PAGE gels and transferred onto nitrocellulose blotting membranes (Millipore). The membranes were then blocked with 5% bovine serum albumin and incubated with specific primary antibodies diluted in 5% BSA overnight at 4 °C. Then the antigen–antibody complexes were visualized using the enhanced chemiluminescence detection system (Millipore, Billerica) in accordance with the manufacturer's instructions. The primary antibodies used are as follows: Anxa8 (1:3,000, ab111708, abcam), Runx2 (1:1,000, ab236639, abcam), and Col1a1 (1:1,000, ab260043, abcam). Horseradish peroxidase-labeled secondary antibody (ab205719, abcam) was used at a 1:5,000 dilution. Then, the antigen–antibody complexes were visualized using the enhanced chemiluminescence detection system (Millipore, Billerica) in accordance with the manufacturer's instructions.

### Alkaline phosphatase staining

Osteoblast cells were washed with PBS and fixed with 70% ethanol for 30 min. The fixing solution was added to the cells and incubated together for 2 min. After incubation, the cells were washed with PBST. Then the cells were stained with ALP substrate solution (ab242287, abcam) and incubated for 25 min at 37 °C. The reaction was stopped by washing the cells with PBS, and the purple color was imaged with light mciroscopy.

### Dual-luciferase reporter assay

The constructs containing lncTUG1 3′ UTR were cotransfected with miR-22-5p mimic or a negative control (miR-NC) into the osteoblast cells by using Lipofectamine 3000 (Invitrogen). After 48 h, cells were harvested, and luciferase activities were measured by the Dual-Luciferase Reporter Assay Kit (Promega). The relative luciferase values were normalized to the Renilla luciferase value. The expression of each target gene was calculated from the ratio between the firefly and renilla (internal control) luciferase activities following the instruction of the kit.

### RNA pull-down

Cells transfected with biotinylated miRNA mimics or negative control (50 nM) using Lipofectamine RNAiMAX Transfection Reagent (13,778,150, Invitrogen) were harvested 48 h post-transfection. Following sonication, remaining cell lysates were incubated with C-1 magnetic beads (Life Technologies) at 4 °C for 2 h and then purified using RNeasy Mini Kit (QIAGEN, Duesseldorf, Germany) for analysis.

### RNA immunoprecipitation

Osteoblast cells were lysed using Magna RIP RNA-binding protein immunoprecipitation kit (17–700, Millipore) following the manufacturer's instructions. Then, the cells were incubated with the RIP buffer containing magnetic beads coated with Ago2 antibodies (Millipore). IgG was paralleled as a negative control. After 2 h of incubation at 4 °C, the coprecipitated RNAs were extracted by TRIzol kit (Invitrogen). The immunoprecipitated RNAs were detected by qRT-PCR.

### Quantitative real-time PCR

Total RNA was extracted from the osteoblasts using TRIzol reagent (Invitrogen) following the manufacturer's instructions. Complementary DNA was obtained from a 1 μg RNA sample. qRT-PCR was then performed using the standard SYBR Green Assay protocol on the ABI PRISM 7500 Sequence Detection System and specific primers. GAPDH was used as a control for normalization. The primer sequences were as follows: 5’-TCACATAGCTCTGCCAATCG-3’ (forward [F]), 5’-AGGATGGGATTCATGCTCAG-3’ (reverse [R]) for Anxa8; 5’-GAGCTGCACTGACCAGTAGG-3’ (F), 5’-GTGCTGGCAGATGGATCACT-3’ (R) for miR-22-5p; 5′-GGGAAACTGTGGCGTGAT-3′ (F), 5′-GAGTGGGTGTCGCTGTTGA-3′ (R) for GAPDH. The relative expression levels were calculated using DataAssist software (Applied Biosystems) using the formula 2^−ΔΔCt^.

### Establishment of a mouse model of bone fracture healing

The mouse model of closed femoral fractures was generated as described previously.[12] C57BL/6 J mice (male, 8–10 weeks, 25–30 g) were purchased from Vital River Laboratories (Beijing Vital River Laboratory Animal Technology Co., Ltd.) and were fed in cages in a standard environment with adequate water, food, ventilation, and 12 h light. Animal ethics was approved by the Institutional Animal Care and Use Committee of Shandong Provincial Hospital (20210309-02). The surgery was performed under general anesthesia and in sterile condition. A small incision was made at medial knee, and a hole was drilled at the intercondylar notch with an 18-gauge needle (Terumo). A Kirschner wire was inserted into the right femoral bone marrow cavity. After incision was sutured, a closed fracture was produced at the midshaft of the right femur using a fracture machine (three-point bending), with a metal dull blade (weighted 500 g) dropping from a height of 35 cm, and X-ray was taken to confirm the fracture. All mice were equally and randomly assigned into three groups after the surgery: control (ctl), oe-NC, and oe-lncTUG1. The fractures were injected in situ twice a week according to the assigned groups. Radiographic imaging was used to monitor the progression of callus formation, bony union, and subsequent remodeling of the bony callus. Bone healing outcomes were evaluated using an arbitrary grading system: 0 point–no sign of healing; 1 point–callus formation; 2 points–partial healing the fracture; 3 points–disappearance of the fracture line; and 4 points–complete fracture healing.

### Hematoxylin and eosin staining

Tissue samples were hydrated in distilled water. The Hematoxylin, Mayer’s (Lillie’s Modification) solution in the H&E Stain kit (abcam) was added to completely cover tissue section and incubated for 5 min. Excess stain on the samples was removed by rinsing with two changes of distilled water. Bluing Reagent was then added to completely cover tissue section and incubated for 15 s. After rinsing with distilled water, the slides were dipped in absolute alcohol. Eosin Y Solution (Modified Alcoholic) was added to completely cover tissue section to excess and incubated for 3 min. The slides were rinsed with absolute alcohol after incubation and dehydrated in three changes of absolute alcohol. Finally, the slides were clear and mounted in synthetic resin.

### Statistical analysis

Statistical analysis was performed using the SPSS 18.0 software. Data were expressed as mean ± standard deviation. Differences between more than two groups were calculated with the one-way analysis of variance test, and differences between two groups were compared by t test. All experiments were performed in triplicate. The significance level was set at *p* < 0.05.

## Results

### The exosomes derived from BMSCs promote the activity of osteoblasts

**Fig. 1:**
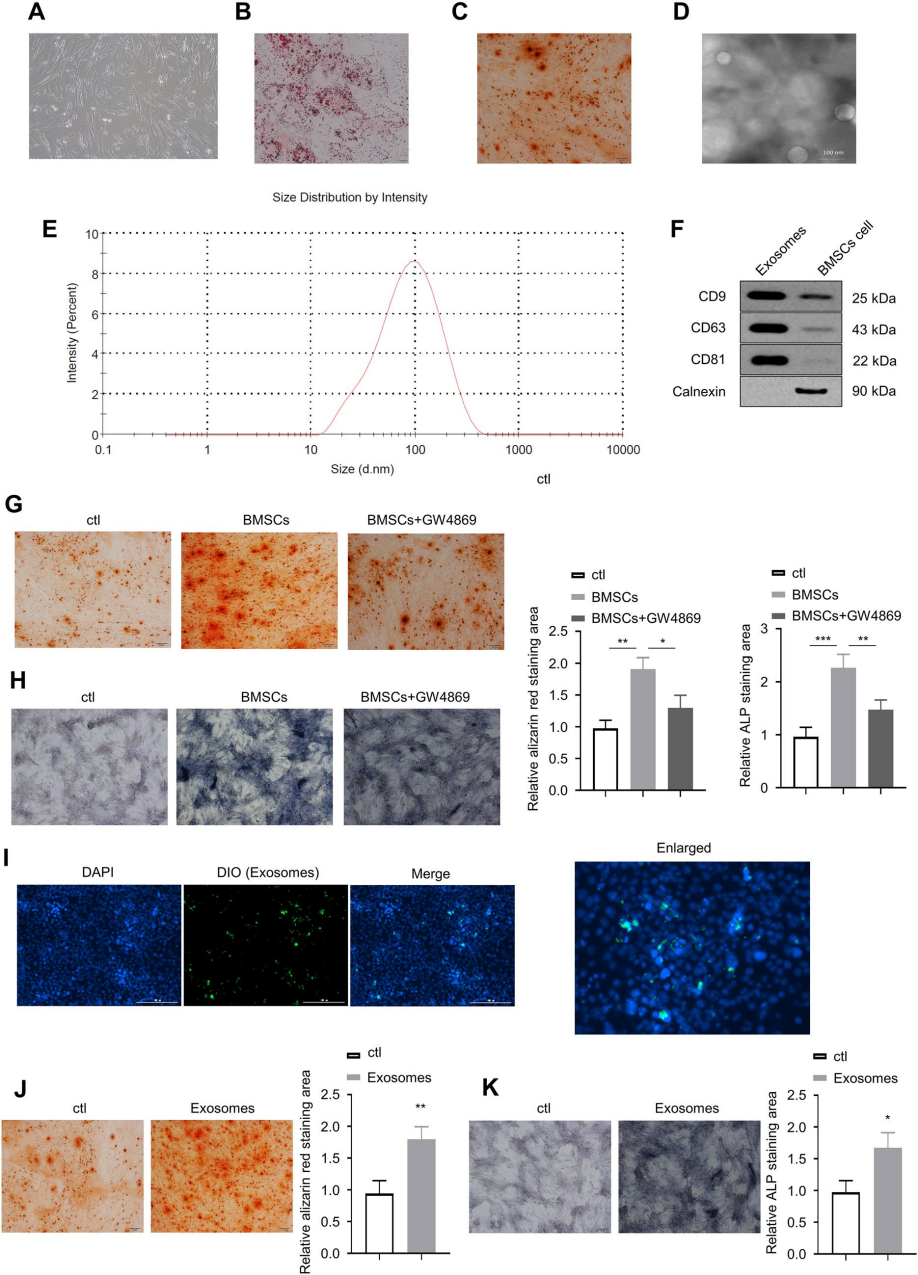
The exosomes derived from BMSCs promote the activity of osteoblasts. **A** BMSCs are spindle shaped. **B** Oil red O staining of BMSCs. Red indicates lip droplets. **C** Alizarin red S staining of BMSCs. Red indicates calcium nodules. **D** BMSCs have an oval shape with apparent bilayer membranes and **E** 100 nm in diameter. **F** Expression of CD9, CD63, CD81, and HSP70 in exosomes and BMSCs. **G** Alizarin red S staining of osteoblasts after co-culture with BMSCs or BMSCs + GW4869. Red indicates calcium nodules. **H** ALP staining of osteoblasts after co-culture with BMSCs or BMSCs + GW4869. Larger purple area indicates a higher level of alkaline phosphatase activity. **I** Fluorescence microscopy image of the phagocytosis of exosomes by osteoblasts. **J** Alizarin red S staining of osteoblasts after co-culture with BMSC-derived exosomes. **K** ALP staining of osteoblasts after co-culture with BMSC-derived exosomes. *p < 0.05, **p < 0.01, ***p < 0.001

BMSCs were isolated from mouse femur and tibia and cultured for the following experiments. After 72 h, we observed the cells under a light microscope (100x), and the images revealed that BMSCs are spindle-shaped (Fig. 1A). Third-generation BMSCs were obtained and cultured for 3 weeks to induce adipogenic and osteogenic differentiation. The cultured cells were then stained with either Oil red O or alizarin red S. Using a light microscope at 400 × magnification, we observed lipid droplets and calcium nodules in respective cells (Fig. 1B, C). Exosomes derived from BMSCs have an oval shape with apparent bilayer membranes and 100 nm in diameter (Fig. 1D, E). Western blotting (WB) analyses showed high expression of CD9, CD63, CD81, and HSP70 but no expression of calnexin in exosomal samples, whereas BMSC protein samples had a high expression of calnexin with no expression of CD9, CD63, CD81, or HSP70 (Fig. 1F). BMSCs and mouse osteoblasts were co-cultured in transwell chambers for 2 weeks, and the osteoblasts were stained using either alizarin red S or alkaline phosphatase (ALP, purple) staining solutions. We quantified and compared the staining areas in the BMSC co-culture group to those in the control group (ctl) and to those in the BMSCs with exosome-inhibitor GW4869 group (BMSCs + GW4869) at 400 × magnification using the light microscope. The co-culture group had a significantly larger alizarin red staining area (i.e., more calcium nodules) than ctl (*p* < 0.01) as well as BMSCs + GW4869 (*p* < 0.05; Fig. 1G). Similarly, a significantly larger ALP staining area was seen in the co-culture group compared with that in ctl (*p* < 0.001) and to that in BMSCs + GW4869 (*p* < 0.01; Fig. 1H). We labeled exosomes with lipophilic green, fluorescent dye (DiO) and incubated them with osteoblasts, of which the nuclei were stained with 4′,6-diamidino-2-phenylindole (DAPI), for fluorescence microscopy study. We detected the phagocytosis of exosomes by osteoblasts, as demonstrated in Fig. 1I. Further, the isolated and cultured BMSC-derived exosomes were co-cultured with osteoblasts for 2 weeks, and then the osteoblasts were stained with alizarin red S. More red calcium nodules were found in the co-culture group relative to the control using the light microscope at 200 × magnification (*p* < 0.01; Fig. 1J). Similarly, response to ALP staining was more prominent in the co-culture group compared with that of the control (*p* < 0.05; Fig. 1K).

### BMSC-derived exosomal LncTUG1 promotes osteoblast activity

**Fig. 2:**
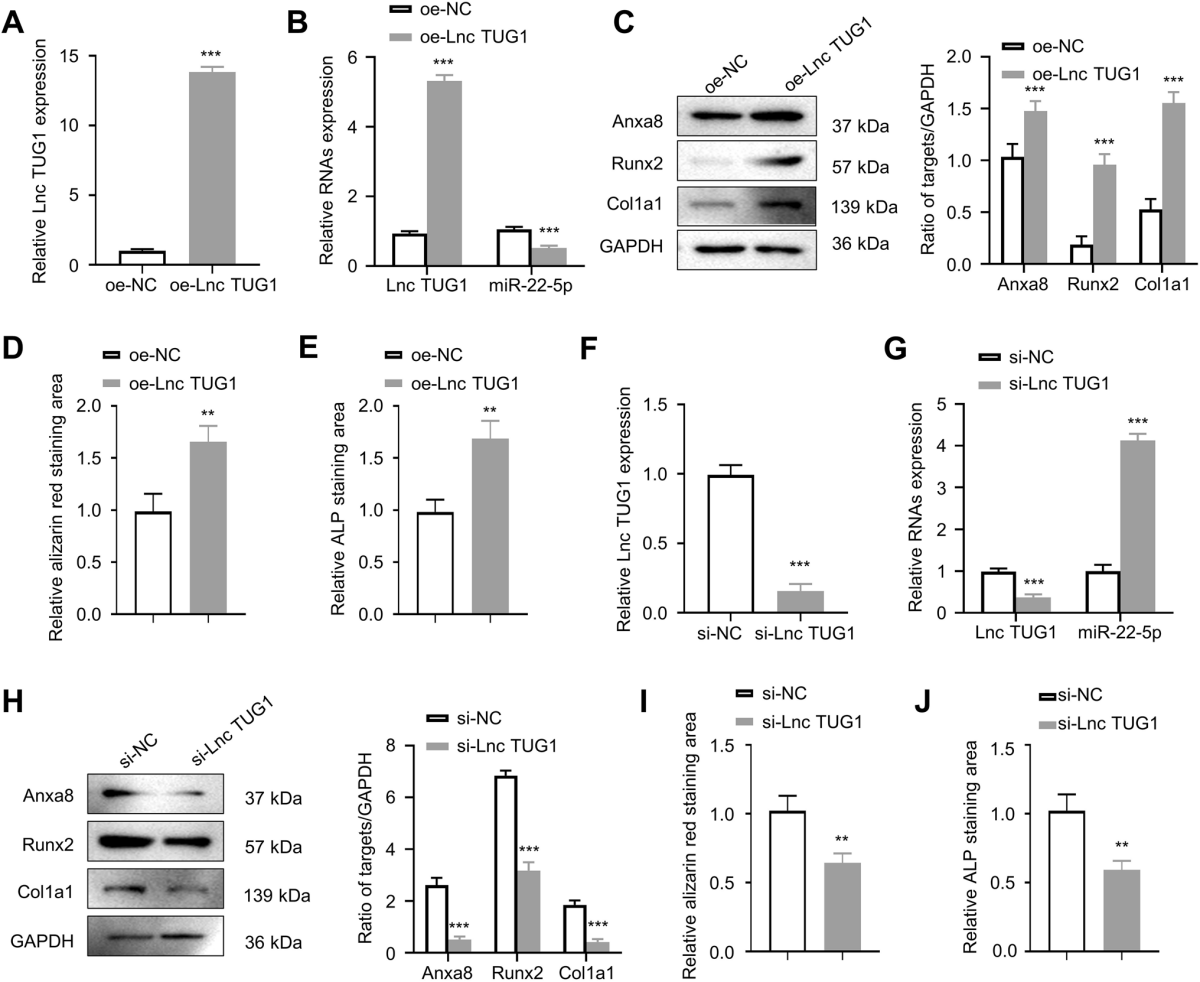
BMSC-derived exosomal lncTUG1 promotes osteoblast activity. **A** Relative lncTUG1 expression in exosomes derived from oe-NC- and oe-lncTUG1-transfected cells. **B** Relative lncTUG1 and miR-22-5p expressions in osteoblasts after co-culture with either the oe-NC or oe-lncTUG1 exosomes. **C** Expression of Anxa8, Runx2, and Col1a1 in osteoblasts after co-culture with either the oe-NC or oe-lncTUG1 exosomes. **D** Alizarin red S staining of osteoblasts after 2 weeks of osteogenic induction. **E** ALP staining of osteoblasts after 2 weeks of osteogenic induction. **F** LncTUG1 expression in si-NC- and si-lncTUG1-treated osteoblasts. **G** Expression levels of lncTUG1 and miR-22-5p in osteoblasts after silencing lncTUG1 in BMSC-derived exosomes. **H** Expression of Anxa8, Runx2, and Col1a1 in si-NC- and si-lncTUG1-treated osteoblasts. **I** Alizarin red S and **J** ALP staining of si-NC- and si-lncTUG1-treated osteoblasts after 2 weeks of osteogenic induction. **p < 0.01, ***p < 0.001

Exosomal samples were collected from overexpressed lncTUG1 (oe-lncTUG1)- or negative control (oe-NC)-treated BMSC. The oe-lncTUG1 and oe-NC exosomes were then co-cultured with osteoblasts, respectively, and qRT-PCR was performed to analyze their lncTUG1 and miR- 22-5p expression levels. The relative expression of lncTUG1 was significantly higher in oe-lncTUG1 than oe-NC; on the contrary, miR-22-5p was significantly reduced in oe-lncTUG1 samples compared with that in oe-NC samples (*p* < 0.001; Fig. 2A, B). WB results showed that Anxa8, Runx2, and Col1a1 had a higher expression in oe-lncTUG1 samples than that in oe-NC (Fig. 2C). Following 2 weeks of osteogenic induction, osteoblasts that had been co-cultured with the exosomes were stained with either alizarine red S or ALP. The results of both experiments showed that the oe-lncTUG1 group had a significantly larger staining area (*p* < 0.01; Fig. 2D, E). In the following loss-of-function experiments through lncTUG1 knockdown, we detected a significant increase in miR-22-5p expression (*p* < 0.001; Fig. 2F, G) but marked reductions in Anxa8, Runx2, and Col1a1 expressions (Fig. 2H) compared with that in negative control-transfected samples (si-NC). The alizarine red and ALP staining areas were smaller in osteoblasts after silencing of lncTUG1 relative to si-NC (*p* < 0.01; Fig. 2I, J). Overall, the results of the gain- and loss-function assays suggest that lncTUG1 in exosomes derived from BMSC can promote osteoblast activity.

### LncTUG1 can target miR-22-5p, and increasing miR-22-5p can reverse the osteopromoting effect of increased lncTUG1

**Fig. 3:**
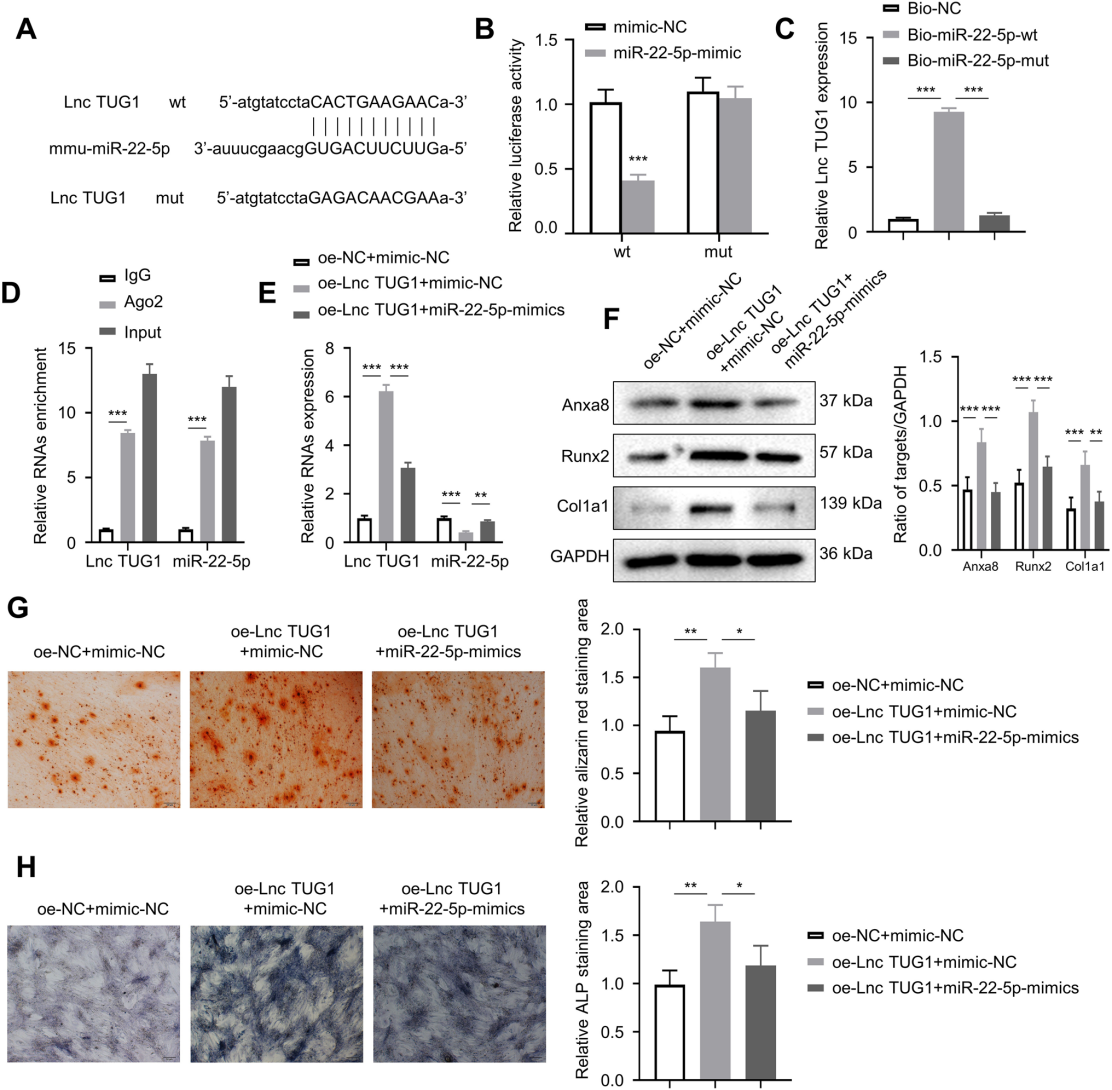
LncTUG1 can target miR-22-5p and increasing miR-22-5p can reverse the osteopromoting effect of increased LncTUG1. **A** The predicted binding sites between lncTUG1 and miR-22-5p. **B** Relative luciferase activity of wide-type (wt) and mutant (mut) lncTUG1 3′ UTR-treated cells that were transfected with either mimic-NC or miR-22-5p. **C** Relative lncTUG1 expression of cells transfected with Bio-NC, Bio-miR-22-5p-wt, or Bio-miR-22-5p-mut. **D** Relative enrichment of lncTUG1 and miR-22-5p in Ago2 and IgG RIP samples. **E** Relative expression of lncTUG1 and miR-22-5p in cells transfected with oe-NC + mimic-NC, oe-lncTUG1 + mimic-NC, and oe-lncTUG1 + miR-22-5p-mimics. **F** Expression of Anxa8, Runx2, OCN, Col1a1 in cell transfected with oe-NC + mimic-NC, oe-lncTUG1 + mimic-NC, and oe-lncTUG1 + miR-22-5p-mimics. **G** Alizarin red S staining of osteoblasts after two weeks of osteogenic induction. Red indicates calcium nodules. **H** ALP staining of osteoblasts after two weeks of osteogenic induction. Larger purple area indicates a higher level of alkaline phosphatase activity. *p < 0.05, **p < 0.01, ***p < 0.001

Figure 3A illustrates the predicted binding sites between lncTUG1 and miR-22-5p. To confirm whether lncTUG1 interacts with miR-22-5p, we conducted a wide-type (wt) and mutant (mut) lncTUG1 3′ UTR luciferase reporter vector. Co-transfection with wt lncTUG1 and miR-22-5p mimics significantly reduced luciferase activity (*p* < 0.001), whereas co-transfection with mut lncTUG1 and miR-22-5p mimics resulted in no change in luciferase signal (Fig. 3B). miR-22-5p mimics transfection appears to be able to repress the activity of wt lncTUG1 in osteoblasts. Further, we performed an RNA pull-down assay, in which biotinylated miR-22-5p-wt (bio-miR-22-5p-wt), biotinylated miR-22-5p-mut (bio-miR-22-5p-mut), and NC (bio-NC) were transfected into osteoblasts. LncTUG1 expression of samples in the bio-miR-22-5p-wt group was significantly higher than that of the bio-miR-22-5p-mut and that of bio-NC (Fig. 3C). We then carried out an RNA immunoprecipitation assay (RIP) with an argonaute 2 (Ago2) antibody. Both lncTUG1 and miR-22-5p were enriched in Ago2 RIP samples, compared with IgG RIP samples, suggesting that lncTUG1 can interact with miR-22-5p (*p* < 0.001; Fig. 3D). Moreover, we analyzed the relative expression of lncTUG1 and miR-22-5p in exosomes derived from samples treated with either oe-NC + mimic-NC, oe-lncTUG1 + mimic-NC, or oe-lncTUG1 + miR-22-5p-mimics. The real-time reverse transcription PCR (qRT-PCR) assays revealed that the oe-lncTUG1 + mimic-NC group had a significantly higher expression of lncTUG1 (*p* < 0.001) but lower expression of miR-22-5p (*p* < 0.001 or 0.01) relative to the oe-NC + mimic-NC and oe-lncTUG1 + miR-22-5p-mimics groups (Fig. 3E). We also analyzed the protein expression of Anxa8, Runx2, OCN, Col1a1 in these three groups. Compared with oe-NC + mimic-NC group, oe-lncTUG1 + mimic-NC samples showed significantly increased expression levels of the above four proteins (*p* < 0.01; Fig. 3F). Furthermore, two weeks after the induction of osteogenesis, mineralized nodular structures were present and positively stained with either alizarine red S or ALP. For both staining experiments, the oe-lncTUG1 + mimic-NC group had a significantly larger staining area relative to the oe-NC + mimic-NC (*p* < 0.001) and oe-NC + mimic-NC groups (*p* < 0.05; Fig. 3G, H).

### miR-22-5p targets Anxa8, and knockdown of Anxa8 reverses the osteogenic effect of miR-22-5p inhibitors

**Fig. 4:**
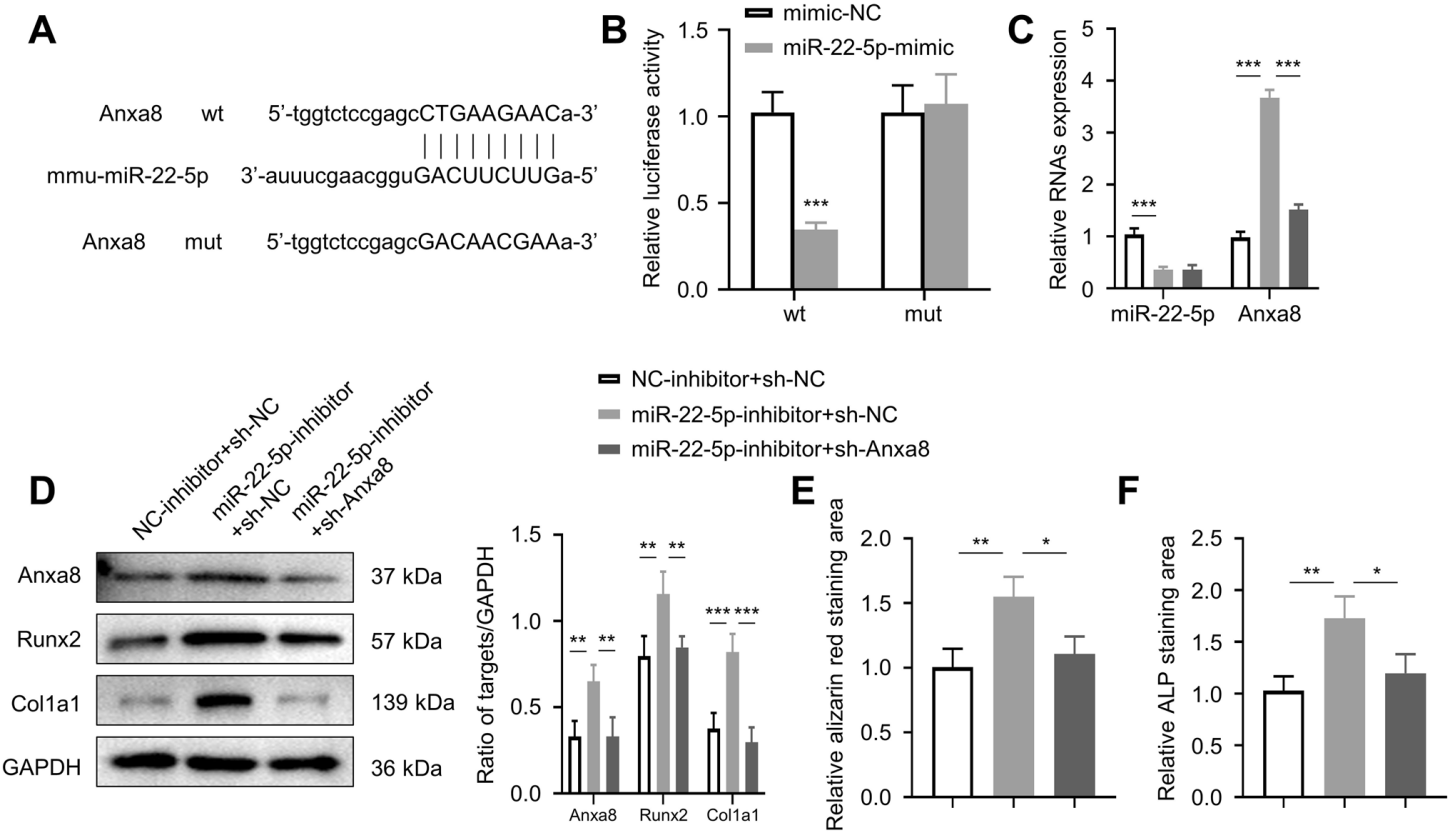
miR-22-5p targets Anxa8, and knockdown of Anxa8 reverses the osteogenic effect of miR-22-5p inhibitors. **A** The predicted binding sites between miR-22-5p and Anxa8. **B** Relative luciferase activity of wt Anxa8 and mut Anxa8 cells that were transfected with either miR-22-5p or NC mimics. **C** Relative expression of miR-22-5p and Anxa8 in NC-inhibitor + sh-NC, miR-22-5p-inhibitor + sh-NC, and miR-22-5p-inhibitor + sh-Anxa8 osteoblasts. **D** Expression of Anxa8, Runx2, and Col1a1 in the three groups of osteoblasts. **E** Alizarin red S staining of osteoblasts after two weeks of osteogenic induction. **F** ALP staining of osteoblasts after two weeks of osteogenic induction. *p < 0.05, **p < 0.01, ***p < 0.001

Anxa8 is a protein that functions as an anticoagulant that indirectly inhibits the thromboplastin-specific complex, which is involved in the blood coagulation cascade. Figure 4A illustrates the predicted binding sites between miR-22-5p and Anxa8. To confirm whether miR-22-5p can interact with Anxa8, we used a dual-luciferase reporter assay. Co-transfection with wt Anxa8 and miR-22-5p mimics led to a significantly lower fluorescence value compared to that of the wt Anxa8 and mimic-NC group (*p* < 0.001), but there was no difference in the fluorescence value between the two comparison groups that had been transfected with mut Anxa8 (Fig. 4B). For all the following experiments, there were three comparison groups: NC-inhibitor + sh-NC (Group 1), miR-22-5p-inhibitor + sh-NC (Group 2), and miR-22-5p-inhibitor + sh-Anxa8 (Group 3). qRT-PCR analyses showed that Group 2 had significantly lower miR-22-5p expression relative to Group 1 (*p* < 0.001); Group 1 and Group 3 had significantly reduced expression of Anxa8 than compared with that of Group 2 (*p* < 0.001; Fig. 4C). WB results revealed that Anxa8, Runx2, and Col1a1 expressions in Group 3 were significantly higher relative to Group 1 but significantly lower relative to Group 2 (*p* < 0.01; Fig. 4D). Moreover, alizarin red S staining and ALP activity analyses showed that Group 3’s staining areas were significantly larger relative to Group 1 (*p* < 0.01) but smaller relative to Group 2 (*p* < 0.05; Fig. 4E, F).

### BMSC-exos promote the protein expression of Anxa8 in osteoblasts, lncTUG1–miR-22-5p–Anxa8 constitutes a ceRNA regulatory mechanism

**Fig. 5:**
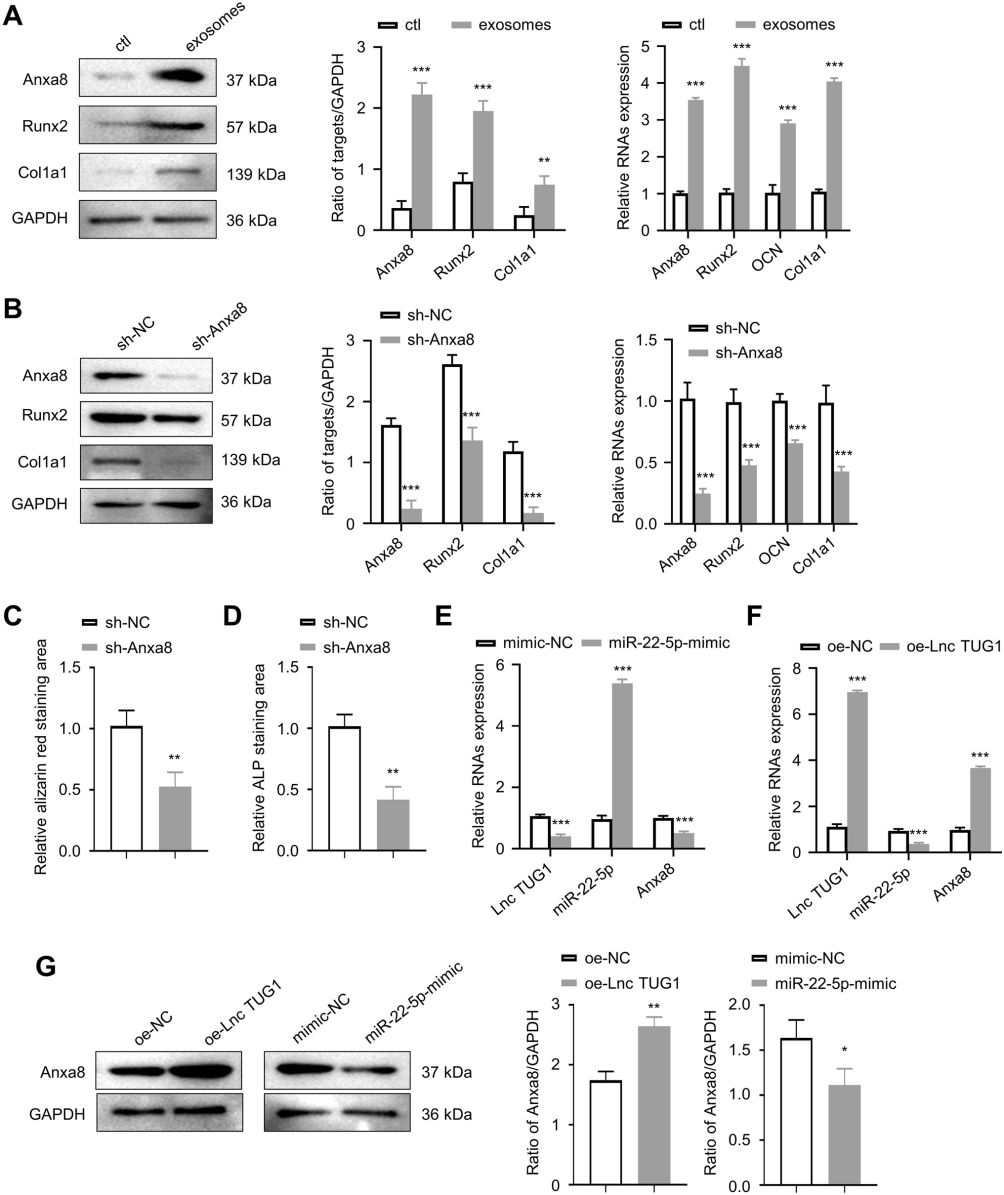
BMSC-Exos promote the protein expression of Anxa8 in osteoblasts, lncTUG1–miR-22-5p–Anxa8 constitutes a ceRNA regulatory mechanism. **A** Expression of Anxa8, Runx2, and Col1a1in BMSC-derived exosomes (BMSC-Exos). **B** Expression of Anxa8, Runx2, and Col1a1in BMSC-Exos treated with either sh-NC or sh-Anxa8. **C** Alizarin red S staining of osteoblasts from the sh-NC and sh-Anxa8 groups after two weeks of osteogenic induction. **D** ALP staining of osteoblasts osteoblasts from the sh-NC and sh-Anxa8 groups after two weeks of osteogenic induction. **E** Relative lncTUG1, miR-22-5p, and Anxa8 expressions in mimic-NC- and miR-22-5p-mimic-treated cells. **F** Relative lncTUG1, miR-22-5p, and Anxa8 expressions in oe-NC- and oe-lncTUG1-treated cells. **G** Expression of Anxa8 in oe-NC, oe-lncTUG1, mimic-NC, and miR-22-5p-mimic groups. **p < 0.01, *** p < 0.001

We then explored the mechanisms by which lncTUG1, miR-22-5p, and Anxa8 mediate the observed effects above. BMSC-Exo samples had higher expressions of Anxa8, Runx2, and Col1a1 relative to controls (*p* < 0.001; Fig. 5A). Following lentiviral knockdown of Anxa8 in the osteoblasts, Anxa8, Runx2, OCN, and Col1a1 expressions reduced significantly compared to that of controls (*p* < 0.001; Fig. 5B). Two weeks after staining with alizarin red S, the sh-Anxa8 group showed fewer red calcium nodules than the sh-NC group (*p* < 0.01; Fig. 5C), and the ALP activity also decreased in the sh-Anxa8 group (*p* < 0.01; Fig. 5D). Next, the osteoblasts were transfected with miR-22-5p mimics, and the expressions of lncTUG1, miR-22-5p, and Anxa8 were detected by qRT-PCR. Compared with the mimic-NC group, miR-22-5p-mimic samples showed significantly higher expression of miR-22-5p but lower expressions of lncTUG1 and Anxa8 (Fig. 5E). Similarly, after lentiviral transfection up-regulated lncTUG1 (oe-lncTUG1) in the osteoblasts, the expression levels of lncTUG1, miR-22-5p and Anxa8 detected by qRT-PCR. In the oe-lncTUG1 group, lncTUG1 and Anxa8 expressions increased (*p* < 0.001) but miR-22-5p expression reduced (*p* < 0.001) than those in the oe-NC group (Fig. 5F). After osteoblasts were treated with oe-lncTUG1 or miR-22-5p mimics, the expression of Anxa8 protein was detected by WB. Compared with controls, Anxa8 expression increased in the oe-lncTUG1 group but decreased in the miR-22-5p-mimic group (*p* < 0.01; Fig. 5G).

### Upregulated lncTUG1 promotes the fracture recovery in mice

**Fig. 6:**
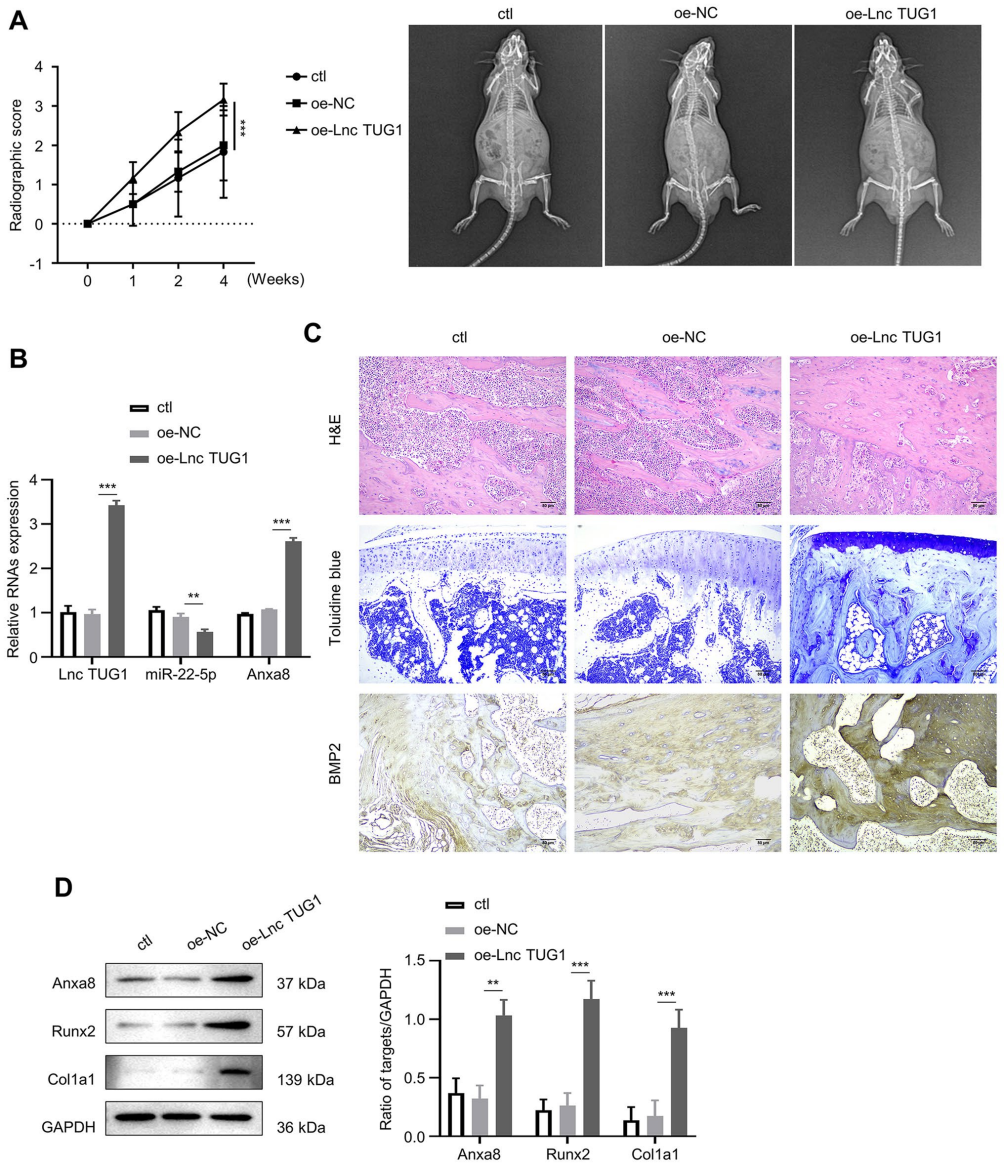
Upregulated lncTUG1 promotes the fracture recovery in mice. **A** Radiographic score of ctl, oe-NC, and oe-lncTUG1 groups. **B** Relative lncTUG1, miR-22-5p, and Anxa8 expression in tissues obtained from the ctl, oe-NC, and oe-lncTUG1 mice. **C** Hematoxylin and eosin, Toluidine Blue, and bone morphogenetic protein 2 staining of fracture tissues obtained from the ctl, oe-NC, and oe-lncTUG1 mice. **D** Expression of Anxa8, Runx2, and Col1a1 in fracture tissues. **p < 0.01, ***p < 0.001

To determine whether lncTUG1 affects osteoblast activity in vivo, we established a mouse model of closed femoral fractures. Bone healing outcomes were evaluated using an arbitrary grading system: 0 point–no sign of healing; 1 point–callus formation; 2 points–partial healing the fracture; 3 points–disappearance of the fracture line; and 4 points–complete fracture healing. Radiographic analyses showed that the scores of each group gradually increased over time. Notably, at Week 4, there was no difference between the ctl and oe-NC groups, but a significant difference was seen between the oe-lncTUG1 and oe-NC groups (Fig. 6A; *p* < 0.001). The expression levels of lncTUG1, miR-22-5p and Anxa8 in the fracture tissue of each group were detected by qRT-PCR. In the oe-lncTUG1 mice, lncTUG1 and Anxa8 expression were significantly higher but miR-22-5p was lower than the oe-NC mice (Fig. 6B; *p* < 0.001). At Week 2, fracture tissues were obtained from each group and stained with hematoxylin and eosin (HE), Toluidine Blue (TB), bone morphogenetic protein 2 (BMP-2) solutions. Compared with the ctl and oe-NC groups, the oe-lncTUG1 group showed higher levels of osteoblasts and BMP-2 (Fig. 6C; *p* < 0.01). In addition, WB detected the expressions of Anxa8, Runx2, and Col1a1 were the highest in the fracture tissue of the oe-lncTUG1 group (Fig. 6D; *p* < 0.01).

## Discussion

Bone fracture healing is a complex physiologic process that involves changes in the expression of several thousand genes [2]. Aberrant gene expression may lead to abnormal or delayed healing that increases patients’ risk of bone and related complications. Our understanding of bone regeneration and fracture repair at the cellular and molecular levels has advanced greatly over the past decades. Characterization of the genes and proteins involved in the process of bone healing may lead to the identification of novel therapies that can be used as adjuncts or alternatives to traditional bone regeneration treatments. LncRNAs, a highly heterogeneous group of transcripts that regulate gene expression, have been shown to be a key regulator of bone development and homeostasis [13]. In this study, we explored the role of an aberrantly expressed LncRNA, lncTUG1, in fracture healing. We found that BMSC-derived exosomal lncTUG1 could inhibit miR-22-5p activity, leading to an increase in the expression of Anxa8 in osteoblast cells, which results in enhanced osteoblast activity and bone regeneration. Our findings suggest that there is a lncTUG1–miR-22-5p–Anxa8 ceRNA regulatory network involved in the process of bone fracture recovery, providing new insight into the regulatory mechanisms of bone development and repair.

The role of lncRNAs in bone formation and bone regeneration has been increasingly recognized [14, 15]. Multiple MSC-associated lncRNAs have been shown to regulate osteogenic differentiation, which are reviewed elsewhere [16–19]. The proper activity of these lncRNAs is essential to key cellular processes of fracture healing, and changes in their expression can alter diverse molecular pathways and lead to disturbed bone regeneration. LncRNAs can act as scaffolds, decoys, signals, and guides; they are key regulators of gene expression in a wide range of biological contexts, including epigenetic transcriptional control, splicing, translation, and protein stability [7]. Briefly, in the regulation of osteogenesis, lncRNAs may function as either competitive endogenous RNAs for miRNAs or as microRNA sponges; combine with RNA-binding protein to change the expression of osteogenesis-related genes; recruit chromatin-modifying complexes to or directly modulate the promoter of osteogenesis-related genes; and act as an antisense transcript whose transcription can activate or inactivate the sense gene [15].

Despite the importance of lncRNA expression in bone reconstruction and bone regeneration, the specific biological role, and functional aspects of the vast majority of the identified fracture healing-associated lncRNAs remain largely unknown [5, 20]. The present study showed that lncTUG1–miR-22-5p–Anxa8 constituted a ceRNA regulatory network. LncTUG1 seems to be able to bind to miR-22-5p, acting as RNA sponges to block the binding of miR-22-5p to their target sites on Anxa8. Similar findings were reported by previous studies. For instance, according to Chen et al*.*, KCNQ1OT1 could modulating miR-701-3p and its target gene, fibroblast growth factor receptor 3, through which this lncRNA accelerated fracture healing [21]. These findings highlight the role of interplay between lncRNAs and miRNAs in the molecular and cellular events during the fracture healing cascade.

The present study provided the first insight into the functions and mechanisms of this novel lncRNA in fracture healing, and its results supported the functionality of lncTUG1. In addition to in vitro work, we also demonstrated the promoting effect of increased lncTUG1 on fracture recovery in vivo. In the animal experiment, the extent of healing differed significantly with the optimal result seen in the oe-lncTUG1 group, but future in vivo investigations are required to confirm this finding. Our current understanding of the expression pattern of lncTUG1 in different phases of fracture healing, and its biological functions and mechanisms of action is still limited. Further research is warranted to determine if this lncRNA has the potential to aid in the development of biomarkers or of novel gene therapies for fracture healing.

In conclusion, the results of the present study show that lncTUG1 from BMSCs may be transported into osteoblasts via exosomes, and increased lncTUG1 in osteoblasts may upregulate Anxa8 expression via reducing the inhibiting action of miR-22-5p on Anxa8, which ultimately leads to enhanced osteoblast differentiation and activity. The lncRNA–miRNA pathway is the most frequently reported at present but of note, accumulating evidence points to a sophisticated and multi-layered mode of regulation by lncRNAs in osteogenic differentiation. Future research is needed to expand our understanding of how lncRNAs and miRNAs cooperate with each other and how their interactions affect gene expression at the transcriptional and post-transcriptional levels that influences the fracture healing process.

## Supplementary Information

Below is the link to the electronic supplementary material.

Supplementary file1 (DOCX 2666 KB)

## Data Availability

The datasets during and/or analysed during the current study are available from the corresponding author on reasonable request.
