# Supplementary material for: Bone marrow mesenchymal stem cells derived exosomal Lnc TUG1 promotes bone fracture recovery via miR-22-5p/Anxa8 axis
Source: Hum Cell. 2023 Mar 23;36(3):1041–53. doi: 10.1007/s13577-023-00881-y (PMC10110643; doi:10.1007/s13577-023-00881-y)
Supplement: Supplementary file 1 — Supplementary file1 (DOCX 2666 KB) [file 13577_2023_881_MOESM1_ESM.docx]

Figure 1F





CD9





CD63





CD81





Calnexin

Fig2C





Anxa8





Runx2





Col1a1





GAPDH

Figure 2H


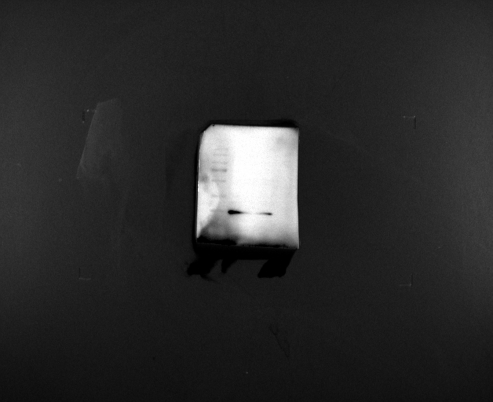


Anxa8





Col1a1





Runx2





GAPDH

Figure 3F





Anxa8








Col1a1

Runx2





GAPDH

GAPDH

Col1a1

Figure 4D





Anxa8





Runx2





Col1a1





GAPDH

Figure 5A





Anxa8





Runx2





Col1a1





GAPDH

Figure 5B





Anxa8





Runx2







Col1a1





GAPDH

Figure 5G





Anxa8 Left





GAPDH Left





Anxa8 Right





GAPDH Right

Figure 6D





Anxa8





Runx2





Col1a1





GAPDH
